# Loss of O6‐Methylguanine‐DNA Methyltransferase Protein Expression by Immunohistochemistry Is Associated With Response to Capecitabine and Temozolomide in Neuroendocrine Neoplasms

**DOI:** 10.1002/wjs.12471

**Published:** 2025-01-17

**Authors:** Mark G. Evans, Joanne Xiu, Sourat Darabi, Anthony Crymes, Adam Bedeir, David A. Bryant, Matthew J. Oberley, Michael J. Demeure

**Affiliations:** ^1^ Departments of Pathology and Clinical and Translational Research Caris Life Sciences Phoenix Arizona USA; ^2^ Precision Medicine Program Hoag Family Cancer Institute Newport Beach California USA; ^3^ Department of Medicine, Keck School of Medicine University of Southern California Los Angeles California USA; ^4^ Basis Phoenix High School Phoenix Arizona USA; ^5^ Translational Genomics Research Institute Phoenix Arizona USA

**Keywords:** capecitabine, MGMT immunohistochemistry, neuroendocrine tumors, temozolomide

## Abstract

**Background:**

A recent prospective phase II study (ECOG‐ACRIN E2211) demonstrated that MGMT deficiency was associated with a significant response to capecitabine and temozolomide (CAPTEM) in pancreatic neuroendocrine neoplasms (NENs); however, routine MGMT analysis in NENs was not recommended. Our study sought to demonstrate whether loss of MGMT protein expression is associated with improved overall survival (OS) in patients receiving CAPTEM for NENs from various tumor sites.

**Materials and Methods:**

Paraffin‐embedded tumor samples were evaluated by immunohistochemistry (IHC) using an MGMT monoclonal antibody. Intact MGMT protein expression (i.e., IHC positivity) was defined as any staining intensity (> 1+) in ≥ 36% of neoplastic cells according to an internal validation study. IHC and pyrosequencing for *MGMT* promotor methylation was performed in an independent cohort of 58 NENs. Real‐world OS was extrapolated from insurance claims data with Kaplan–Meier estimates from the date of first CAPTEM administration to the last date of contact.

**Results:**

The study cohort included 80 patients (42 men and 38 women) with a median age of 57 years (range: 19–89). They had various NENs (33 pancreatic, 17 intestinal, 7 pulmonary, 8 other, and 15 of unknown origin) treated with CAPTEM. The median OS for the 48 patients with MGMT negative tumors was 31 months compared to 17.5 months for the 32 patients whose tumors were MGMT positive by IHC (HR: 1.75 [95% CI: 1.066–2.87] and *p* = 0.025). IHC results from the independent cohort of 58 NENs showed only 57% concordance with pyrosequencing results.

**Conclusions:**

MGMT promotor status by IHC may be a clinically useful indicator that predicts improved OS for NENs treated with CAPTEM, but IHC does not reliably correlate with the findings of *MGMT* promoter methylation by pyrosequencing.

## Graphical abstract

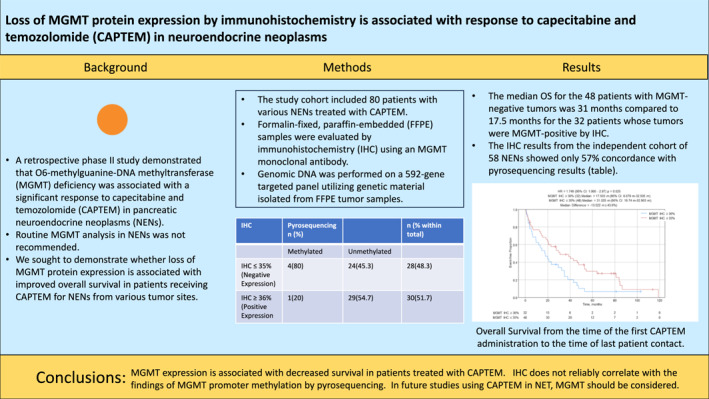

## 1 Introduction

Temozolomide (TEM) has been utilized in the treatment of advanced‐stage neuroendocrine neoplasms (NENs), either alone or in combination with other therapies, and has been formally recommended as a monotherapy for pancreatic, thoracic, and midgut NENs [1, 2, 3, 4]. It is an oral alkylating agent that is responsible for O6 guanine methylation, which potentiates DNA damage and promotes tumor cell destruction. However, this outcome is reversed by the enzyme O6‐methylguanine‐DNA methyltransferase (MGMT). The activity of MGMT is suppressed by promoter methylation in some tumors such as gliomas, rendering alkylating treatments more effective. In patients with NENs, promoter methylation appears to be less frequent, but as immunohistochemistry (IHC) in NENs frequently shows MGMT protein loss, other mechanisms may be responsible for decreased MGMT expression [5]. Ultimately, the antimetabolite capecitabine (CAP) has been added to TEM regimens as a method of synergistic suppression of MGMT activity.

An initial study investigating the use of CAPTEM was limited to a retrospective analysis of 30 patients diagnosed with pancreatic NENs [6]. It reported a median progression‐free survival (PFS) of 18 months and a risk reduction of 70%. This work prompted an effort to investigate TEM in the treatment of NENs by the NCI Neuroendocrine Tumor Clinical Trials Planning Meeting [7]. The findings of the eventual prospective phase II study, ECOG‐ACRIN E2211, demonstrated a superior median PFS of 22.7 months in 72 patients treated with CAPTEM for pancreatic NENs, compared to a median PFS of 14.4 months for the 72 individuals receiving monotherapy TEM [5]. Moreover, this research included an assessment of impaired MGMT activity by IHC or promoter methylation studies; MGMT deficiency as demonstrated by either technique was ultimately associated with the response rate to CAPTEM by response evaluation criteria in solid tumors (RECIST). Furthermore, this study was restricted to pancreatic neuroendocrine cancers and, notably, did not demonstrate whether the IHC and pyrosequencing results were comparable in the same tumor samples.

There are multiple treatment options for advanced NENs, including but not limited to peptide receptor radiotherapy, sunitinib, everolimus, levatinib, and CAPTEM [8], but little evidence guiding selection or recommending tumor sequencing exists for these therapies. Given the potential role of MGMT as a biomarker for NEN therapy selection, we sought further to explore its impact on the effectiveness of TEM‐combination regimens. Specifically, we wanted to assess the role of MGMT deficiency in predicting CAPTEM response. Since IHC is a readily available diagnostic methodology at most hospitals and laboratories and can be performed considerably less expensively than other molecular studies, such as promoter methylation analysis, we focused on the use of an IHC assay.

## 2 Materials and Methods

### 2.1 Study Cohort

We queried all formalin‐fixed paraffin‐embedded (FFPE) NEN samples submitted by oncologists to a commercial CLIA‐certified laboratory (Caris Life Sciences, Phoenix, AZ, USA) for the purposes of comprehensive genomic sequencing and biomarker testing for eventual treatment selection in their clinical practice. For inclusion in this study, the tumors must have undergone IHC testing for MGMT, and the patients from which the samples were obtained had to have received CAPTEM according to clinical data obtained from insurance claims, through a commercial administrative database subscription, which encompass detailed records of health services, including prescribed medications, procedures performed, and established diagnoses. We documented sample collection sites, and those that matched the primary site designated by the submitting institution were regarded as primary tumors, whereas all samples obtained from sites different from the annotated primary site were considered metastatic lesions. This research was conducted in accordance with guidelines of the U.S. Common Rule, Declaration of Helsinki, and Belmont Report. As consistent with policy 45 CFR 46.101(b), the study was performed using retrospective deidentified clinical data and patient consent was not required.

### 2.2 Next‐Generation Sequencing (NGS)

Genomic DNA was input into a previously described 592‐gene targeted panel [9] utilizing genetic material isolated from FFPE tumor samples. Sequencing was completed using the NextSeq platform (Illumina, Inc., San Diego, CA, USA). A custom‐designed SureSelect XT assay was used to enrich 592 whole‐gene targets (Agilent Technologies, Santa Clara, CA, USA). The sequencing detected variants with > 99% confidence based on allele frequency and amplicon coverage, with an average sequencing depth of coverage of > 500 and an analytic sensitivity of 5%. Matched normal tissue was not sequenced.

Genetic variants identified were interpreted by board‐certified molecular geneticists as described previously [10]. Tumor mutational burden (TMB) was measured by counting all nonsynonymous missense, nonsense, in‐frame insertion/deletion, and frameshift mutations found per tumor that had not been previously described as germline alterations in dbSNP151 and Genome Aggregation Database (gnomAD) databases or benign variants identified by Caris geneticists. In accordance with the KEYNOTE‐158 pembrolizumab trial [11], high TMB (TMB‐H) was defined by a cut‐off of 10 mutations/megabase (mut/Mb). Caris Life Sciences was a participant in the Friends of Cancer Research TMB Harmonization Project [12].

### 2.3 Microsatellite Instability (MSI‐H)/Mismatch Repair (MMR) Protein Status

A comparison of two methodologies was used to determine the MSI‐H or MMR status of the tumors profiled, including IHC (MLH1, M1 antibody; MSH2, G2191129 antibody; MSH6, 44 antibody; and PMS2, EPR3947 antibody [Ventana Medical Systems, Inc., Tucson, AZ, USA]) and NGS (> 2800 target microsatellite loci were aligned with the hg38 reference genome from the University of California, Santa Cruz Genome Browser database). The results were highly concordant between the two methodologies as reported previously [13].

### 2.4 MGMT IHC

IHC was performed on complete sections of FFPE tissues mounted on glass slides, which were stained using mouse anti‐MGMT, clone MT23.2 (Invitrogen, Thermo Fisher Scientific, Waltham, MA, USA) with automated staining techniques, as per the manufacturer's instructions, and were optimized and validated as per CLIA/CAP and ISO requirements. Intact MGMT protein expression (i.e., IHC positivity) was designated as any staining intensity (≥ 1+) in ≥ 36% of neoplastic cells. An internal validation study established this threshold by comparing test samples to known positive and negative tissues classified according to IHC intensity scores reported by The Human Protein Atlas or RNA levels documented by the European Bioinformatics Institute.

### 2.5 MGMT Promoter Methylation Pyrosequencing

DNA extracted from FFPE tumor samples was treated with bisulfite and underwent PCR amplification specific to *MGMT* exon 1 (GRCh37/hgl9—chromosome 10: 131,265,448–131,265, and 560), followed by pyrosequencer‐based analysis of 5 CpG sites (CpGs 74–78) using the PyroMark system (QIAGEN, Germantown, MD, USA). Hypermethylation consisted of ≥ 9% methylation, with ≥ 7% to < 9% being equivocal.

### 2.6 Patient Outcomes and Statistics

Real‐world overall survival (OS) was determined from insurance claims data and was defined as the first CAPTEM administration to the date of the patient's last known clinical activity. In cases with no insurance claims for a period exceeding 100 days, it was inferred that the patient had expired. Conversely, patients with a documented clinical activity within 100 days prior to the latest data update were censored in the analysis. Kaplan–Meier survival estimates were generated for cohorts defined by IHC characteristics. Hazard ratios (HRs) were computed utilizing the Cox proportional hazards model, and significant differences in survival times were assessed with the log‐rank test, where *p* < 0.05 was considered significant. Chi‐squared/Fisher’s exact tests were applied elsewhere as appropriate, with *p*‐values adjusted for multiple comparisons (*p* < 0.05).

## 3 Results

### 3.1 Cohort and MGMT IHC Results With Comparison to MGMT Promoter Methylation Testing

**FIGURE 1:**
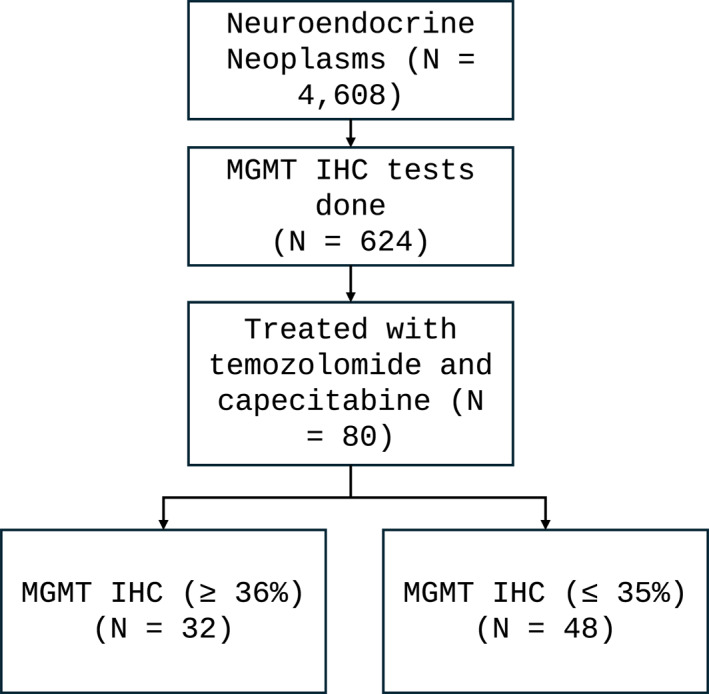
Strobe diagram outlining the study cohort derived from neuroendocrine neoplasm samples submitted to Caris Life Sciences.

Among the 4608 NEN samples submitted to Caris Life Sciences for testing, 80 patients (42 men, 38 women, and median age: 57 years [range: 19–89]) receiving CAPTEM had specimens submitted for testing that included MGMT IHC with 32 samples being IHC positive and the remaining 48 being negative [Figure 1]. The tumors arose in various primary sites (33 pancreatic, 17 intestinal, 7 pulmonary, 8 other, and 15 of unknown origin), and 72.5% (58/80) of the tested samples were derived from metastatic lesions. Patient/tumor characteristics are provided in Table 1, with additional tested sample details available in Supporting Information S1 Table 1.

**TABLE 1:** Patient and NEN tumor characteristics.

		MGMT IHC ≥ 36%	MGMT IHC ≤ 35%	Total	*p*‐value
Age (years)	Age range	33–89	19–83		0.53
	Age median	57.5	57		
Sex	Female	17	21	38	0.68
	Male	15	27	42	
Pathologic diagnosis	NEC	10	18	28	0.71
	NET, G1	9	14	23	
	NET, G2	13	15	28	
	NET, G3		1	1	
Primary tumor site	Pancreas	10	23	33	0.21
	Small intestine	5	4	9	
	Lung	3	4	7	
	Colon	4		4	
	Rectum	3	1	4	
	Stomach	1	1	2	
	Thymus		2	2	
	Bladder		1	1	
	CNS		1	1	
	Nasopharynx		1	1	
	Pelvis		1	1	
	Unknown	6	9	15	
Specimen sites	Primary	10	12	22	0.38
	Metastasis	22	36	58	0.18
	Brain		1	1	
	Breast	2		2	
	Flank		1	1	
	Liver	14	25	39	
	Lymph nodes		4	4	
	Mesentery	1		1	
	Neck		1	1	
	Orbit	1		1	
	Pancreas		1	1	
	Presacral region		1	1	
	Rectum	1		1	
	Rib	1		1	
	Sigmoid colon	1		1	
	Skin of flank		1	1	
	Spine	1		1	
	Spleen		1	1	
Median follow‐up time		82.3 months	81.3 months	82.3 months	0.97

An independent cohort of 58 NEN samples without available clinical or treatment data included 30 tumors positive for MGMT IHC, with the remaining 28 being negative. Of the IHC negative specimens, only four demonstrated increased *MGMT* promoter methylation, and one IHC positive tumor showed evidence of hypermethylation by pyrosequencing.

### 3.2 Biomarker and Mutation Associations Detected by NGS

**FIGURE 2:**
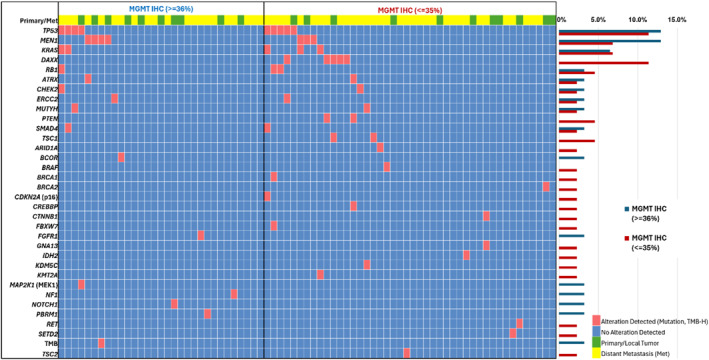
Oncoprint diagram with pathogenic and likely pathogenic mutations (single nucleotide variants and insertions–deletions) and TMB‐H status categorized by MGMT IHC result for primary and metastatic NENs; right bar diagrams represent the prevalence of genetic changes compared between the MGMT IHC groups.

Certain mutations were more frequently observed in MGMT IHC negative tumors, particularly those involving genes that regulate the cell cycle, such as *DAXX* and *CDKN2A* [Figure 2], which were altered in only primary tumor specimens. Of note, biomarkers such as *TSC1*/2, *BRAF*, *IDH2,* and *RET* were mutated exclusively in the MGMT IHC negative group. *TP53*, *KRAS*, and *RB1* alterations were observed frequently in both IHC groups, with the latter two genes being affected exclusively in primary tumor samples. Of note, none of the specimens analyzed showed evidence of microsatellite instability. Only one tumor with MGMT IHC positivity demonstrated TMB‐H.

### 3.3 Patient Outcomes

**FIGURE 3:**
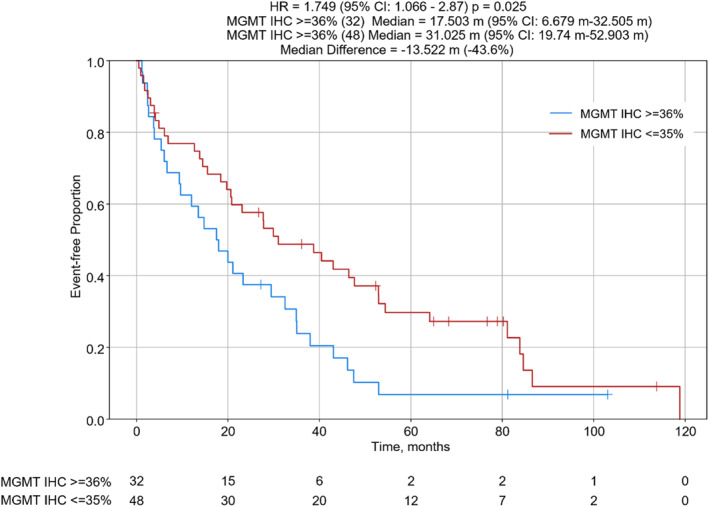
OS from the time of first CAPTEM administration to the time of last patient contact comparing NENs with MGMT IHC ≥ 36% (*N* = 32) versus MGMT ≤ 35% (*N* = 48); CI, confidence interval; HR, hazard ratio; and m, months.

**FIGURE 4:**
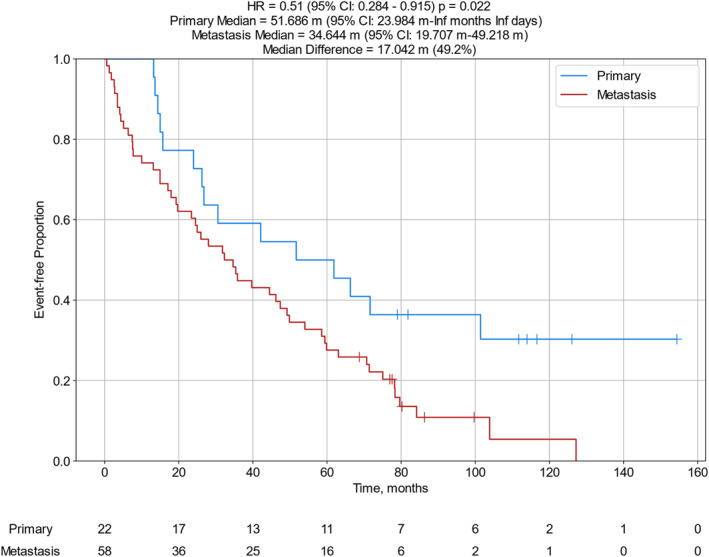
OS from the time of specimen collection to the time of last patient contact comparing samples obtained from primary tumor sites (*N* = 22) versus metastatic lesions (*N* = 58); CI, confidence interval; HR, hazard ratio; inf, infinite; and m, months.

**FIGURE 5:**
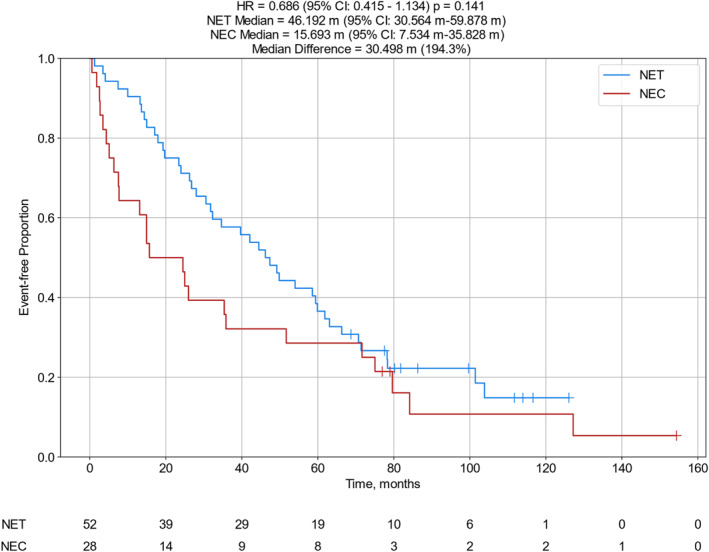
OS from the time of specimen collection to the time of last patient contact comparing tumors diagnosed as NET, G1‐3 (*N* = 52) versus NEC (*N* = 28); CI, confidence interval; HR, hazard ratio; and m, months.

For patients with clinical information available, all patients were diagnosed with Stage IV disease at the time of sample testing. Their tumors were classified as poorly differentiated neuroendocrine carcinomas (NECs) or more differentiated neuroendocrine tumors (NETs), grades 1–3 (G1‐3) [Table 1 and Supplementary Table S1] as per the 2022 WHO Classification of Neuroendocrine Neoplasms [14]. The median OS for the 48 patients with MGMT IHC negative tumors was 31.0 months compared to 17.5 months for the 32 patients whose tumors were MGMT positive by IHC (hazard ratio (HR): 1.75 [95% confident interval (CI): 1.066–2.87] and *p* = 0.025) [Figure 3]. Similar analysis comparing samples obtained from primary tumors versus metastatic lesions revealed improved OS to CAPTEM in those obtained from primary tumor sites (median 51.7 vs. 36.4 months, HR 0.51 [95% CI: 0.284–0.915], and *p* = 0.022) [Figure 4]. Those specimens diagnosed as NET (G1–3) had longer OS than those classified as NEC, although the differences were not statistically significant (median 46.2 vs. 15.7 months, HR 0.686 [95% CI: 0.415–1.13), and *p* = 0.141) [Figure 5]. Moreover, nonsignificant differences in OS were calculated when subtype analyzes were performed comparing tumors arising from specific organs or at certain anatomic sites. Median follow‐up time was 82 months [Table 1].

## 4 Discussion

Our study suggests that the MGMT methylation status by IHC could serve as a clinically useful biomarker to consider when selecting CAPTEM for the treatment of NENs arising from various anatomic sites. Specifically, we demonstrated that MGMT deficiency, as evidenced by a lack of expression by IHC, was associated with significantly improved OS compared to those tumors with retained MGMT activity. This observation is added to a growing body of literature that presents mixed results regarding MGMT's ability to predict the efficacy of CAPTEM [15, 16, 17, 18]. Many of these investigations were small studies, often focused on pancreatic NENs, and relatively high overall response rates to CAPTEM independent of the MGMT status obscured their findings. Consistent with our work, one study of 107 NENs from several origins were treated with alkylating agents in 53 cases, discovering that PFS and OS from first treatment administration were significantly higher in tumors demonstrating loss of MGMT activity [19]. Moreover, the first large prospective phase II study of TEM in patients with advanced pancreatic neuroendocrine tumors, ECOG‐ACRIN E2211, showed that a lack of MGMT expression by IHC was associated with a superior overall response rate, irrespective of treatment with either TEM or CAPTEM [5].

In comparison to previous research, our study also demonstrates the variability of MGMT IHC interpretation. Although our work employed an internally validated threshold for positivity as any staining intensity observed in ≥ 36% of tumor cells, previous studies endorsed the use of 0, 5, or 10% positivity cut‐offs for establishing MGMT deficiency [15, 16, 17, 19]. Unfortunately, these lower thresholds may downplay the importance of tumor heterogeneity. We also analyzed our patient cohort according to a cut‐off for positivity of ≥ 1% and did not observe a similar statistically significant result compared to our validated threshold. Ultimately, higher cut‐offs or the use of H‐scoring systems, such as described by Kros et al. [20], may better capture the MGMT status. Clearly, MGMT IHC requires standardization not only with respect to interpretation but also related to staining procedure. Published studies varied in terms of the antibody utilized. Although our analysis incorporated clone MT23.2, similar research reported findings using the clone MT3.1 [16, 17, 20]. Moreover, compared to earlier work, we advocate for using calibrated and automated procedures to avoid staining variability and the use of whole tissue‐mounted slides to visualize possible tumor heterogeneity.

We must also acknowledge that other methods for assessing MGMT deficiency as alternatives to IHC have been explored in NENs. In particular, the ECOG‐ACRIN E2211 study documented that *MGMT* promoter methylation was associated with improved response to CAPTEM [5]. The correlation of the promoter methylation status with TEM efficacy is well‐established in gliomas [21], but its relevance to the treatment of NENs remains unclear and requires further investigation. Moreover, promoter methylation may have limited concordance with IHC as evidenced by several studies [17, 18]. Our personal experience with performing *MGMT* promoter methylation analysis on an independent cohort of 58 NENs revealed only 57% concordance between the expected molecular testing results and MGMT IHC findings. Unfortunately, due to institutional constraints related to sample deidentification, we were unable to complete *MGMT* promoter methylation studies on the CAPTEM‐treated tumors described herein. Instead, our work focuses on IHC as a reliable determinant of MGMT status, and it may prove more readily accessible and cost effective for hospitals and laboratories given the expenses associated with performing molecular studies such as promoter methylation analysis.

The previously discussed studies substantiate the findings of our work, although we must acknowledge several important limitations. Specifically, our study is a retrospective investigation of a modestly sized study cohort confounded by the selection bias of only including patients who received CAPTEM and had tumor samples previously analyzed by MGMT IHC. The included cases comprised a limited representation of specimens from the various anatomic sites and carrying specific NEN diagnoses (i.e., only approximately one‐third were classified as NEC). Given the small subgroup sizes, we could not demonstrate statistically significant differences in OS based on pathologic diagnosis or on primary tumor anatomic sites. Overall, the utility of considering all NENs together may be limited, given the biological differences between these neoplasms arising from various body tissues. Moreover, we are unable to show any concordance of MGMT IHC findings between primary sites and metastatic lesions and its impact on CAPTEM response, as we did not have access to matched specimens obtained from individual patients. Additionally, we could not consider the clinical context (i.e., time to metastasis and evidence of disease progression) whereby metastasis occurred for those cases in which a metastatic lesion was tested. Unfortunately, follow‐up clinical data regarding disease progression or metastasis could not be obtained; given our restricted access to clinical data, with all treatment information being derived from de‐identified insurance claims, we were unable to report dosages received, evidence of partial or complete responses over time (such as evaluation of tumors by radiographic RECIST scores), or therapy‐related toxicity. Moreover, as per the institutional policy, we were unable to contact treating physicians for additional granular details related to follow‐up information involving the patients' tumors and care.

Beyond establishing the role of MGMT as an important biomarker in NENs, our study also demonstrates the landscape of genomic changes present in these tumors. The tested neoplasms featured relatively stable genomes with low TMB that harbored commonly observed driver mutations. Specifically, within metastatic and primary tumor NEN samples, we detected alterations in genes central to DNA‐repair (*CHEK2*, *MUTYH*, and *BRCA2*), the mTOR pathway (*TSC2* and *PTEN*), chromatin remodeling (*MEN1*, *SETD2*, *ATRX*, and *DAXX*), and cell‐cycle regulation (*CDKN2A*) that have been previously implicated in NENs [22, 23, 24, 25]. Moreover, in NECs, we identified known driver mutations in genes such as *RB1*, *TP53*, *KRAS*, and *BRAF* [26, 27, 28]. We also observed that infrequent variants, such as those involving *BRCA2* and *RET*, were seen exclusively within the primary tumors tested. However, we could not definitively explore the reported heterogeneity that can occur between primary and metastatic NEN cells [29], as our study did not analyze matched primary and metastatic specimens from individual patients.

Of note, the genomic changes documented in our study have important outcome and treatment implications. We detected alterations in *DAXX*, *MEN1*, and *ATRX*, with mutations in the latter being only observed in samples extracted from primary tumor sites; these biomarkers have been shown to have prognostic significance in pancreatic NENs [30]. Furthermore, the overall low TMB detected in our cohort could explain the limited utility of immunotherapy in the treatment of NENs [31]. Our work also demonstrated interesting associations with other known cancer‐relevant biomarkers. Particularly, a lack of MGMT expression by IHC was observed along with mutations in *BRAF, BRCA1/2*, *IDH2,* and *RET*, which serve as the targets of important therapies in oncology. Although our study ultimately suggests that MGMT deficiency, as determined by IHC interpretation, may be helpful when selecting CAPTEM for the treatment of NENs, additional investigation is required. We would especially advocate for an evaluation of MGMT status, such as by promoter methylation testing with or without IHC analysis, when conducting CAPTEM‐related clinical trials. Ultimately, the NGS analysis detailed in our work demonstrates utility beyond assessing MGMT, by uncovering co‐occurring clinically relevant biomarker changes that have well‐established treatment implications. Further research into these biomarkers will hopefully uncover new avenues for managing NENs, such as novel combinations incorporating CAPTEM with other targeted therapies.

## Author Contributions

**Mark G. Evans:** data curation, formal analysis, investigation, methodology, project administration, writing—original draft. **Joanne Xiu:** data curation, formal analysis, writing—review and editing. **Sourat Darabi:** conceptualization, data curation, methodology, project administration, writing—review and editing. **Anthony Crymes:** investigation, writing—review and editing. **Adam Bedeir:** writing—review and editing. **David A. Bryant:** investigation, methodology, writing—review and editing. **Matthew J. Oberley:** investigation, methodology, writing—review and editing. **Michael J. Demeure:** conceptualization, formal analysis, funding acquisition, investigation, methodology, project administration, resources, supervision, writing—original draft, writing—review and editing

## Disclosure

M.G.E., J.X., D.A.B., and M.J.O.: Caris Life Sciences (full‐time employment, travel/speaking expenses, and stock/stock options).S.D.: BostonGene (consulting fees). M.J.D.: Lilly, Orphagen, Theralink, Bayer, TD2 OnCusp, Pfizer, Aadi Biosciences, Corcept and Crinetics (consulting fees).

## Ethics Statement

This research was conducted in accordance with guidelines of the U.S. Common Rule, Declaration of Helsinki, and Belmont Report. As consistent with policy 45 CFR 46.101(b), the study was performed using retrospective deidentified clinical data and patient consent was not required.

## Conflicts of Interest

The authors declare no conflicts of interest.

**Abbreviations**
CAP: College of American Pathologists
CAPTEM: capecitabine and temozolomide
CI: confidence interval
CLIA: Clinical Laboratory Improvement Amendments
ECOG‐ACRIN: Eastern Cooperative Oncology Group‐American College of Radiology Imaging Network
FFPE: formalin‐fixed, paraffin‐embedded
gnomAD: Genome Aggregation Database
HR: hazard ratio
IHC: immunohistochemistry
ISO: International Organization for Standardization
m: months
Met: distant metastasis
MGMT: O6‐methylguanine‐DNA methyltransferase
MMR: mismatch repair
MSI: microsatellite instability
mut/Mb: mutations/megabase
NCI: National Cancer Institute
NEC: neuroendocrine carcinoma
NEN: neuroendocrine neoplasm
NET, G1‐3: neuroendocrine tumor, grades 1–3
NGS: next‐generation sequencing
OS: overall survival
PFS: progression‐free survival
RECIST: response evaluation criteria in solid tumors
RNA: ribonucleic acid
TMB: tumor mutational burden
TMB‐H: high tumor mutational burden
WHO: World Health Organization

## Supporting information

Supporting Information S1

Table S1

## Data Availability

The datasets used and/or analyzed during the current study are available from the corresponding author on reasonable request.
