# Supplementary material for: Loss of O6‐Methylguanine‐DNA Methyltransferase Protein Expression by Immunohistochemistry Is Associated With Response to Capecitabine and Temozolomide in Neuroendocrine Neoplasms
Source: World J Surg. 2025 Jan 17;49(4):964–72. doi: 10.1002/wjs.12471 (PMC11994148; doi:10.1002/wjs.12471)
Supplement: Supplementary file 1 — Supporting Information S1 [file WJS-49-964-s002.docx]

**Supplementary Table 1** Additional NEN tested sample details; Abbreviation: CNS – central nervous system
