# Supplementary figures and images for: Loss of O6‐Methylguanine‐DNA Methyltransferase Protein Expression by Immunohistochemistry Is Associated With Response to Capecitabine and Temozolomide in Neuroendocrine Neoplasms
Source: World J Surg. 2025 Jan 17;49(4):964–72. doi: 10.1002/wjs.12471 (PMC11994148; doi:10.1002/wjs.12471)

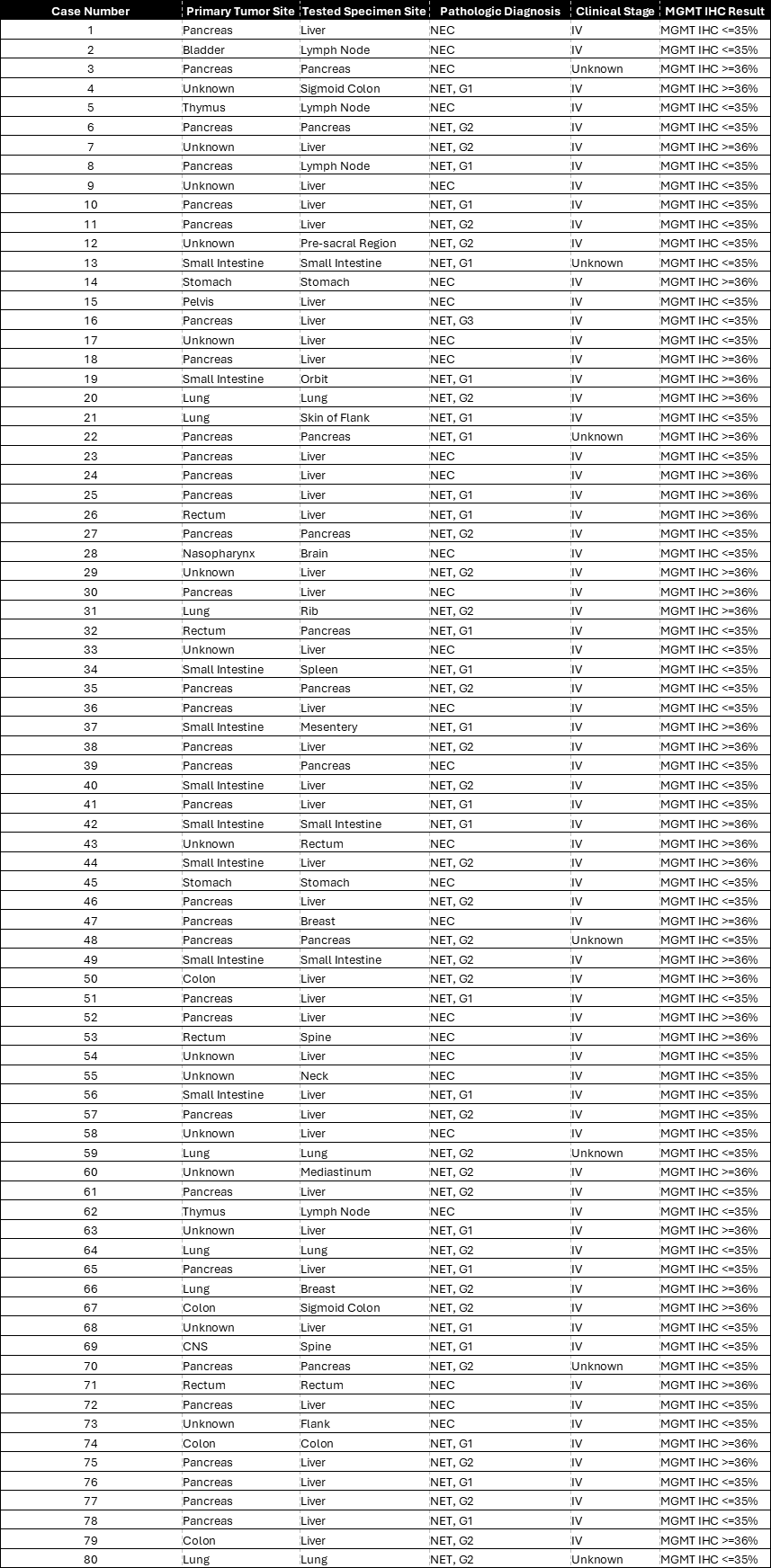

Supplement: Supplementary file 2 — Table S1 [file WJS-49-964-s001.docx]
